# Kilovoltage Energy Significantly Enhances the Therapeutic Efficacy of Low-Dose Radiation in a 3xTg-AD Mouse Model of Alzheimer’s Disease

**DOI:** 10.3390/ijms27125458

**Published:** 2026-06-17

**Authors:** Seungwon Lee, Ye Jin Yoo, Gyehyeong Kim, Eunsu Kim, Subin Yun, Joon Kim, Hoon Ryu, Weonkuu Chung

**Affiliations:** 1 Department of Radiation Oncology, Kyung Hee University College of Medicine, Kyung Hee University Hospital at Gangdong, Seoul 05278, Republic of Korea; swlee00111@gmail.com (S.L.); eunsu2808@naver.com (E.K.); ysb4326@naver.com (S.Y.); junkim91@naver.com (J.K.); 2 Center for Brain Disorders, Brain Science Institute, Korea Institute of Science and Technology (KIST), Seoul 02792, Republic of Korea

**Keywords:** Alzheimer’s disease, low-dose radiation, kilovoltage energy, amyloid-β, tau

## Abstract

Low-dose radiation (LDR) has emerged as a promising therapeutic modality for Alzheimer’s Disease (AD). Although different irradiation protocols have been explored, the optimal parameters for maximizing therapeutic efficacy remain unclear. Radiation energy has been shown to influence radiobiological responses, with more pronounced effects at lower energy ranges. We therefore investigated whether kilovoltage LDR (KLDR) provides superior therapeutic efficacy compared with megavoltage LDR (MLDR) in a murine model of AD(3xTg-AD). To this end, we directly compared the efficacy of MLDR and KLDR in AD model mice to identify an optimal irradiation strategy for LDR treatment with potential relevance to clinical translation in AD. X-rays with 110-kV or 6-MV energy were applied to the brain of AD model mice at an early-stage of disease progression (26–28 weeks age; 0.6 Gy × 5 fractions for 2.5 weeks). After LDR treatment, cognitive function was assessed in AD model mice using passive avoidance (PA) test and novel object recognition (NOR) test. In addition, different molecular markers associated with inflammation, amyloid-beta (Aβ) plaques, tau burden, and neuronal and synaptic degeneration were analyzed in the brain of AD model mice. KLDR (110 kV) significantly inhibited cognitive decline in AD model mice, as demonstrated by both the PA and NOR tests. In addition, KLDR significantly reduced hippocampal levels of GFAP, Iba-1, and pro-inflammatory cytokines (TNF-α, IL-6, and IL-1β), while increasing anti-inflammatory cytokines (TGF-α, TGF-β, and IL-10), and was associated with marked reductions in Aβ and tau levels. Furthermore, the expression levels of Aβ40 and Aβ42 were quantified by ELISA following KLDR and MLDR treatment, revealing a statistically significant reduction in the KLDR group. The degeneration of neurons and synapses was significantly suppressed also at the kilovoltage energy level. Conversely, MLDR (6 MV) exerted minimal effects and did not produce statistically significant improvements. Taken together, our findings demonstrate that radiation energy level is a key determinant of LDR therapeutic efficacy in AD model mice, with KLDR showing significantly greater effectiveness in improving AD-related pathological features than MLDR. Therefore, KLDR may be recommended as a novel radiation protocol for AD treatment.

## 1. Introduction

Low-dose radiation (LDR) therapy attracts public attention with the accumulating evidence for radiation hormesis [1]. Over the past few decades, a great deal of effort has been made to verify the efficacy of LDR in both normal and pathological conditions, finding that LDR plays a key role in triggering multiple adaptive responses in organisms to protect them against endo- and exogenous health threats [2,3,4]. Specifically, regarding transcriptomic analysis, there were two pioneering experiments to assess alterations in gene expression after LDR. Yin et al. reported that 0.1-Gy radiation positively regulated genes engaged in protective and reparative functions in mouse brains, including stress response and DNA synthesis/repair [5]. Lowe et al. showed that 0.1-Gy radiation downregulated cognitive dysfunction-associated neural pathways, and conversely, at a higher dose (2 Gy), any of the pathways were not elicited. Accordingly, the authors proposed that LDR at 0.1 Gy may exert beneficial effects on cognitive function [6]. These results provided a crucial clue that LDR might be used as an alternative modality for improving cognitive decline.

Meanwhile, a previous case-based analysis reported a notable finding: orthovoltage X-rays (typically 150–500 kV) were superior to linear accelerator-based radiotherapy (6 or 10 MV) for the treatment of osteoarthritis. Based on an analysis of 9802 patients reported in the literature, significant clinical benefits of orthovoltage radiotherapy were observed in terms of both complete and partial responses [7]. These findings underscore the significance of radiation energy levels in treating inflammatory and neurodegenerative diseases.

More recently, Kim et al. reported novel findings elucidating part of the mechanism underlying LDR-induced cognitive improvement. They demonstrated LDR-induced microglial polarization from M1 to M2, which in turn upregulated anti-inflammatory cytokines and ultimately mitigated neuroinflammation in Alzheimer’s disease (AD) model mice [8]. These results offered rational evidence of how LDR ameliorates inflammatory milieus in AD brains [9,10]. Furthermore, LDR exerted direct neuroprotective effects by promoting cell viability and reducing DNA fragmentation against β-amyloid (Aβ) toxicity [11]. Clinical studies supported these results, reporting that LDR showed slight or meaningful improvements in early-to-advanced AD patients along with a positive safety profile [12,13,14]. However, the central question of what radiation energy range LDR is most effective for AD treatment has not yet been fully addressed.

Accordingly, the present study conducted a direct comparative evaluation on the efficacy of LDR at two different energy levels—kilovoltage and megavoltage—with respect to improvements in AD pathological features such as inflammation, Aβ/tau burden, neuronal degeneration, and cognitive decline. Eventually, we tried to provide reliable interpretations and rationales for kilovoltage LDR (KLDR), suggesting a novel therapeutic approach for AD. This is the first study of its kind.

## 2. Results

### 2.1. A Marked Inhibition of Cognitive Decline at Kilovoltage, but Not Megavoltage, Energy

**Figure 1:**
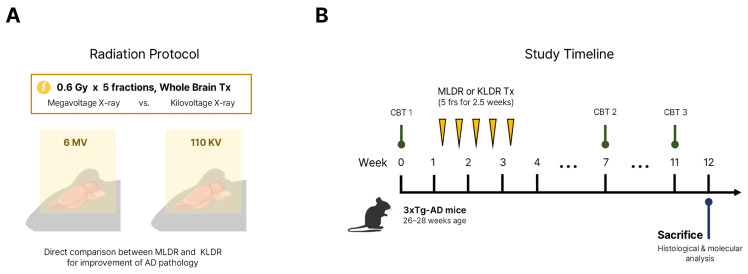
X-ray irradiation protocols (**A**) and experimental schedules (**B**) were summarized. Whole-brain MLDR or KLDR treatments (0.6 Gy × 5 fractions) were performed in 3xTg-AD model mice. Pre- and post-treatment assessments were conducted to evaluate LDR efficacy under two distinct radiation protocols (KLDR vs. MLDR; (**A**,**B**)). Tx, treatment; frs, fractions.

In accordance with the radiation protocol and study timeline, megavoltage LDR (MLDR) or KLDR was delivered to the brains of AD model mice over 2.5 weeks, and then pre- and post-treatment evaluations were carried out using behavioral, histological, and molecular analysis methods (Figure 1A,B).

**Figure 2:**
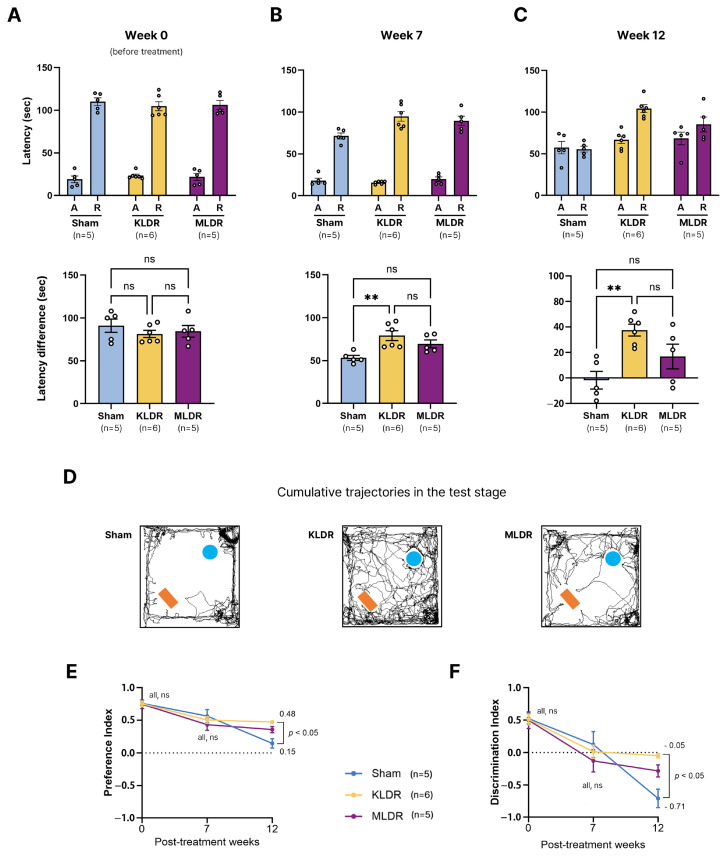
In the PA test, comparable performance was observed among groups at baseline (**A**). KLDR-treated mice showed significantly increased latency at Weeks 7 and 12 compared to the sham control (both, *p* < 0.01), while MLDR had minimal effects (**B**,**C**). In the NOR test, cumulative movement trajectories of all mice during the test stage were visualized, with the blue circle indicating the novel object and the orange rectangle indicating the familiar object (**D**). KLDR mitigated the decline in object interest observed in sham mice, as evidenced by higher PI (**E**) and DI (**F**) values at Week 12 (both, *p* < 0.05). MLDR effects remained negligible across assessments (**E**,**F**). ** denote statistical significance at *p* < 0.01. ns, not significant.

Latency (sec) during the acquisition (A) and retention (R) phases was measured at each time point (Figure 2A–C). The latency difference (R-A) served as an index of memory performance. At baseline (Week 0), the latency difference indicated no significant differences between groups (Figure 2A). Post-treatment, mice in the KLDR group demonstrated significantly delayed entry into the dark chamber compared to sham at Week 7 and Week 12 (both, *p* < 0.01; Figure 2B,C). In addition, novel object recognition (NOR) tests indicated enhanced exploratory trajectories around the novel object (blue circle) in the treatment groups (Figure 2D), with Preference Index (PI) and Discrimination Index (DI) significantly higher in KLDR mice compared to sham at Week 12 (both, *p* < 0.05; Figure 2E,F). In contrast, MLDR treatment produced no significant changes in both tests (Figure 2B,C,E,F). All values were summarized in Supplementary Table S1.

### 2.2. Significant Alterations in Inflammatory and Anti-Inflammatory Marker Levels at Kilovoltage, but Not Megavoltage, Energy

**Figure 3:**
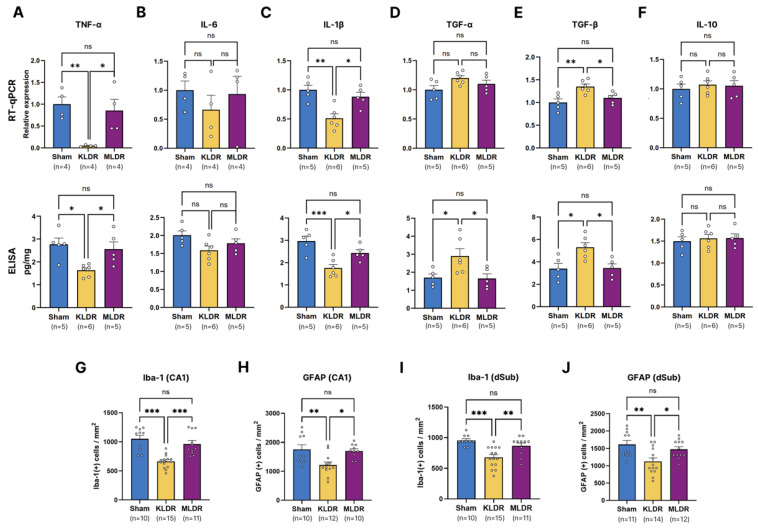
mRNA expression levels of TNF-α, IL-6, IL-1β, TGF-α, TGF-β, and IL-10 were analyzed by RT-qPCR ((**A**–**F**), upper panels), and corresponding protein levels were quantified by ELISA ((**A**–**F**), lower panels). KLDR significantly reduced the expression of the pro-inflammatory cytokines TNF-α and IL-1β while increasing the anti-inflammatory cytokine TGF-β compared with the sham group. Immunohistochemical quantification of Iba-1-positive microglia and GFAP-positive astrocytes in the hippocampal CA1 region and dorsal subiculum (dSub) revealed that KLDR markedly attenuated microgliosis and astrogliosis (**G**–**J**). MLDR showed modest anti-inflammatory effects but was generally less effective than KLDR. *, **, and *** denote statistical significance at *p* < 0.05, *p* < 0.01, and *p* < 0.001, respectively. ns, not significant.

To examine changes in the brain microenvironment after treatment, we first investigated the increase or decrease in the expression levels of inflammatory and anti-inflammatory markers. The expression levels of pro-inflammatory (TNF-α, IL-6, and IL-1β) and anti-inflammatory (TGF-α, TGF-β, and IL-10) cytokines were measured by reverse transcription quantitative polymerase chain reaction (RT-qPCR) and enzyme-linked immunosorbent assay (ELISA). KLDR significantly reduced TNF-α (Figure 3A) and IL-1β (Figure 3C) expression at both the mRNA (upper panel; TNF-α and IL-1β, both *p* < 0.01) and protein (lower panel; TNF-α, *p* < 0.05; IL-1β, *p* < 0.001) levels compared with the sham group, whereas MLDR showed no significant effects. Although IL-6 levels did not differ significantly among the groups, a decreasing trend was observed in the KLDR group at both the mRNA and protein levels (Figure 3B). Among the anti-inflammatory cytokines, KLDR significantly increased TGF-α protein levels (Figure 3D, lower panel; *p* < 0.05) and TGF-β expression at both the mRNA (*p* < 0.01) and protein levels (*p* < 0.05) (Figure 3E) compared with the sham group. However, IL-10 expression was not significantly altered by either treatment (Figure 3F). These results indicate that KLDR, but not MLDR, promotes an anti-inflammatory environment by suppressing pro-inflammatory cytokines and enhancing TGF-α and TGF-β expression. All values were summarized in Supplementary Table S2.

In addition, co-immunostaining experiments revealed reduced Iba-1 and GFAP expressions in the hippocampal CA1 (Figure 3G,H) and dSub (Figure 3I,J) regions after treatment (Supplementary Figure S1A,B). KLDR treatment markedly decreased Iba1-positive and GFAP-positive cell counts in both regions compared to the sham control (Iba-1, *p* < 0.001; GFAP, *p* < 0.01; Figure 3G–J). Conversely, MLDR did not induce significant changes and exhibited markedly lower efficacy compared to KLDR (Iba-1 CA1, *p* < 0.001; Iba-1 dSub, *p* < 0.01; GFAP both regions, *p* < 0.05; Figure 3G–J). All immunofluorescence images and their values were presented in Supplementary Figure S1.

### 2.3. A Marked Reduction in Aβ Deposition at Kilovoltage, but Not Megavoltage, Energy

**Figure 4:**
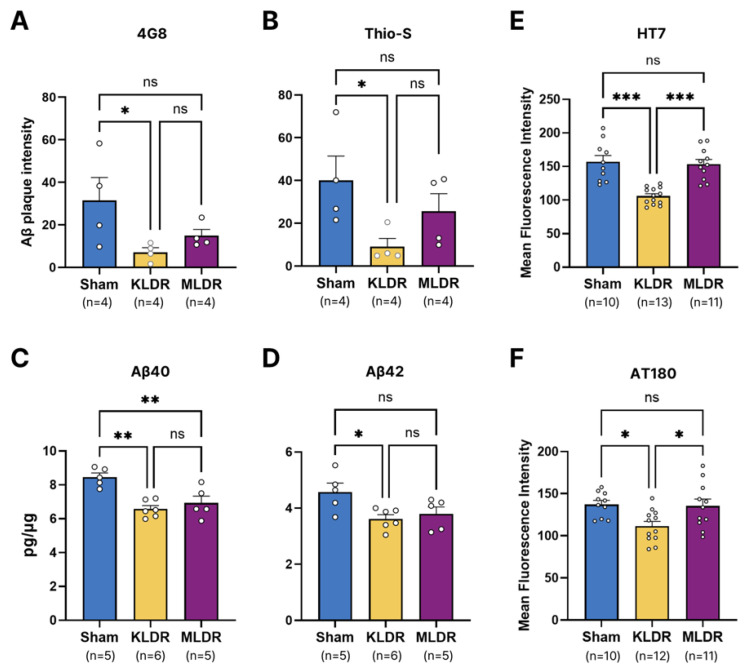
Aβ pathology was evaluated by 4G8 immunostaining (**A**), Thioflavin-S (Thio-S) staining (**B**), and ELISA quantification of soluble Aβ40 (**C**) and Aβ42 (**D**) in the hippocampus. KLDR significantly reduced Aβ plaque burden, Thio-S-positive amyloid deposits, and soluble Aβ40 and Aβ42 levels compared with the sham group. Tau pathology was assessed by immunofluorescence staining for HT7 (**E**) and AT180 (**F**). KLDR markedly decreased the fluorescence intensity of both HT7 and AT180, indicating attenuation of tau accumulation and phosphorylation. In contrast, MLDR produced relatively modest effects on amyloid and tau pathology. *, **, and *** denote statistical significance at *p* < 0.05, *p* < 0.01, and *p* < 0.001, respectively. ns, not significant.

It is well established that Aβ is a critical initiator that greatly affects AD progression via plaque formation. The 4G8 antibody was used to stain Aβ plaques and quantify their intensity in hippocampal slices, finding that Aβ plaque intensity was greatly reduced in the KLDR group (vs. sham, *p* < 0.05; Figure 4A and Supplementary Figure S2A). There was also a drop in the intensity in the MLDR group, which, however, was not statistically significant. Thioflavin S was used to detect Aβ plaques, confirming the reproducibility of the above findings (sham vs. KLDR, *p* < 0.05; Figure 4B and Supplementary Figure S2B). In addition, intracellular Aβ deposition in the amygdala and cortical slices was examined by quantifying 6E10 antibody immunoreactivity. KLDR exerted a pronounced effect in reducing the number of 6E10-positive cells in the slices, whereas MLDR did not elicit these responses (Supplementary Figures S3 and S4). All relevant images and values were presented in Supplementary Figures S2–S4.

Next, Aβ40 and Aβ42 levels were separately quantified using the ELISA technique in the hippocampus of AD model mice. Notably, Aβ42 is known to greatly contribute to Aβ plaque formation. Aβ40 levels were significantly reduced in both KLDR and MLDR groups (vs. sham, *p* < 0.01; Figure 4C). Notably, a significant reduction in Aβ42 levels was observed exclusively in the KLDR group (vs. sham, *p* < 0.05; Figure 4D). A decreasing trend was observed in the MLDR group, but it did not reach statistical significance (Figure 4D). Collectively, KLDR effectively alleviated Aβ burden in both intra- and extracellular regions, whereas MLDR exhibited only minor reductions. All values for the ELISA experiment were summarized in Supplementary Table S3.

### 2.4. A Marked Reduction in Tau Deposition at Kilovoltage, but Not Megavoltage, Energy

Tau is another critical pathogenic protein in AD. Under AD conditions, tau undergoes abnormal phosphorylation, leading to its aggregation into neurofibrillary tangles. In this experiment, tau markers including HT7 (for total tau) and AT180 (for phospho-tau) were identified, and their expression levels were quantified in the hippocampal region of AD model mice (Figure 4E,F and Supplementary Figure S5).

Histological analysis revealed a significant reduction in the fluorescence intensity of HT7 in the KLDR group (vs. sham, *p* < 0.001; Figure 4E and Supplementary Figure S5A). Likewise, the intensity of AT180 was also markedly decreased in the KLDR group (vs. sham, *p* < 0.05; Figure 4F and Supplementary Figure S5B). In contrast, the MLDR group showed no significant reductions in both HT7 and AT180 levels and demonstrated significantly lower efficacy compared to the KLDR group (HT7, *p* < 0.001; AT180, *p* < 0.05; Figure 4E,F). All immunofluorescence images and their values were presented in Supplementary Figure S5.

### 2.5. Significant Inhibition of Neuronal and Synaptic Degeneration at Kilovoltage, but Not Megavoltage, Energy

**Figure 5:**
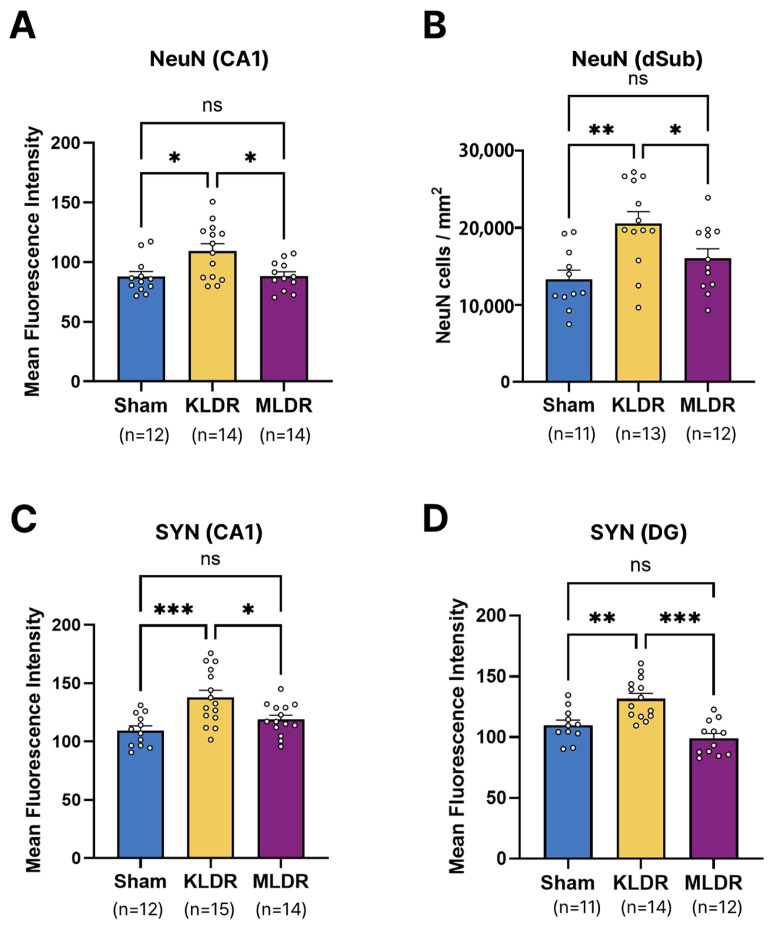
Neuronal survival was assessed by NeuN immunostaining in the hippocampal CA1 region (**A**) and dorsal subiculum (dSub; **B**). KLDR significantly increased NeuN-positive neuronal signals compared with the sham group, indicating enhanced neuronal preservation. Synaptic integrity was evaluated by synaptophysin (SYN) immunostaining in the CA1 region (**C**) and dentate gyrus (DG; **D**). KLDR markedly increased SYN immunoreactivity in both regions, suggesting improved synaptic preservation. In contrast, MLDR exhibited comparatively weaker neuroprotective effects. *, **, and *** denote statistical significance at *p* < 0.05, *p* < 0.01, and *p* < 0.001, respectively. ns, not significant.

Finally, changes in neuronal and synaptic degeneration were examined by quantifying neurons (NeuN) and synaptic function (SYN) markers after KLDR or MLDR treatment. In effect, these two factors are considered to directly impact on cognitive function. NeuN-positive cell counts were measured in the dSub region, but in the CA1 region, NeuN fluorescent intensity was quantified instead of cell counting due to extensive cellular overlap in that area. NeuN fluorescent intensity was significantly increased in the KLDR group (vs. sham, *p* < 0.05 for CA1; Figure 5A). Furthermore, the number of NeuN-positive cells was markedly elevated in the KLDR group (vs. sham, *p* < 0.01 for dSub; Figure 5B and Supplementary Figure S6). Similarly, SYN levels in the CA1 and dentate gyrus (DG) regions were significantly increased in the KLDR group (vs. sham, *p* < 0.001 for CA1, *p* < 0.01 for DG; Figure 5C,D, and Supplementary Figure S7). In contrast, MLDR failed to induce these alterations. Furthermore, significant differences were observed between the KLDR and MLDR groups (*p* < 0.05, *p* < 0.001), indicating that MLDR exerted minimal effects (Figure 5A–D). All immunofluorescence images and their values were presented in Supplementary Figures S6 and S7.

### 2.6. AD-Related Transgene Expression Remains Unchanged After LDR Treatment

The 3xTg-AD mouse model is characterized by the expression of mutant APP, PS1, and Tau transgenes that drive AD-like pathology. To determine whether LDR influences transgene expression, RT-qPCR analysis was performed in the hippocampus. No significant differences were observed in the mRNA levels of human amyloid precursor protein (APP) Swedish mutation (APPswe), human microtubule-associated protein tau (MAPT) P301L mutation (Tau P301L), or presenilin 1 (PS1) among the sham, KLDR, and MLDR groups (Supplementary Figure S8). These findings indicate that LDR treatment did not affect the expression of AD-related transgenes. All values were presented in Supplementary Figure S8.

### 2.7. KLDR Shows Multimodal Actions, Improving Typical AD Pathological Features

**Figure 6:**
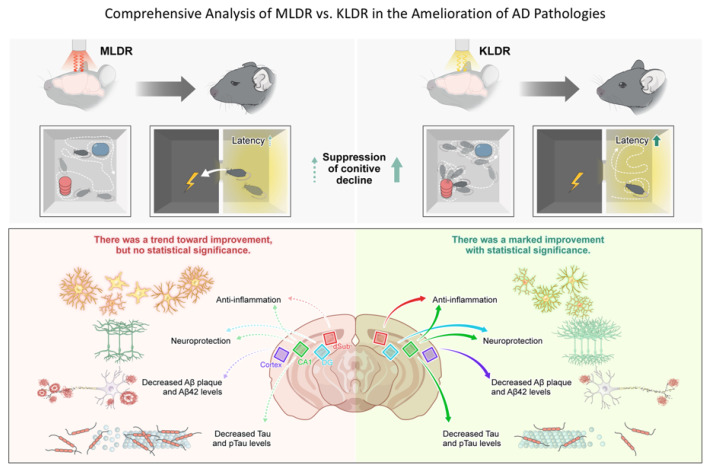
All findings, ranging from cognitive behavioral tests to histological and molecular examinations, are illustrated. Notably, KLDR demonstrated significant improvements in key AD pathological features examined in this study, whereas MLDR exhibited a trend toward improvement that did not reach statistical significance.

All the experiments and results are schematically presented in Figure 6. Low-dose X-rays were applied to the brains of AD model mice at the kilovoltage and megavoltage energy ranges. Cognitive decline was markedly suppressed in the KLDR group. Pronounced anti-inflammatory and neuroprotective effects were also observed at the kilovoltage energy level. Notably, in terms of Aβ and tau burden, mice in the KLDR group exhibited a significant reduction compared to the sham control. In contrast, at the megavoltage energy level, a trend toward improvement of AD pathological features was observed, although it was not statistically significant.

## 3. Discussion

The present study revealed that LDR exerted differential effects in AD model mice depending on the radiation energy level. Specifically, KLDR exerted multimodal actions that markedly alleviated inflammation, intra- and extracellular Aβ accumulation, tau burden, and neuronal and synaptic degeneration in key brain regions involved in cognitive function—namely, hippocampal regions (CA1, DG, dSub), cortex, and amygdala—ultimately leading to a significant suppression of cognitive decline in AD model mice. In contrast, the MLDR regimen exerted only minimal effects on ameliorating these AD pathological features.

Emerging evidence identifies low-energy irradiation as a critical factor in enhancing therapeutic outcomes for neurodegenerative diseases under LDR protocols. Orthovoltage X-rays have demonstrated superior efficacy over megavoltage beams in treating degenerative joint diseases [7]. In addition, Ceyzériat et al. applied 2-Gy 4-MV X-rays to AD model mice over five sessions via a linear accelerator; however, this protocol did not significantly reduce Aβ deposition [15]. Subsequently, they conducted another LDR study using 100 kV X-rays with the same 5 × 2 Gy doses, which significantly reduced Aβ deposition [16]. These findings indicate that LDR efficacy depends on its energy level, with KLDR showing greater therapeutic effects than MLDR in AD model mice.

Neuroinflammation is a common feature of neurodegenerative diseases, having a profound effect on their pathogenesis [17,18]. Importantly, our study found that KLDR has a key role in improving AD-induced inflammatory milieus by markedly suppressing pro-inflammatory cytokine production and attenuating glial activation while promoting anti-inflammatory signaling. Previous studies demonstrated that KLDR could modulate innate immune system by inducing microglial polarization into the M2 profile against lipopolysaccharide (LPS)-induced inflammation, thus suppressing the production of pro-inflammatory cytokines and encouraging that of anti-inflammatory ones [8]. This can be a possible mechanism by which KLDR lessens the production of pro-inflammatory cytokines while promoting the expression of anti-inflammatory cytokines.

In addition, KLDR-induced downregulation of pro-inflammatory cytokines are connected with the reduction in Aβ and tau burden. A previous study showed that interferon-induced transmembrane protein 3 (IFITM3) played a pivotal role in producing Aβ40 and Aβ42 by forming a complex with γ-secretase [19]. Interestingly, IFITM3 expression was governed by pro-inflammatory cytokine levels. In other words, these cytokines positively modulated IFITM3 expression, which in turn increased Aβ production [20,21]. Son et al. reported that 0.1 or 0.3-Gy radiation induced a decrease in IFITM3 expression and then relieved Aβ burden [22]. Meanwhile, it is well established that increased Aβ burden or increased inflammatory responses lead to worsening tau pathology [23,24]. Thus, the KLDR-induced downregulation of pro-inflammatory cytokines is deeply associated with reduction in tau burden. Taken together, it can be speculated that KLDR-induced suppression of pro-inflammatory cytokines leads to reduced IFITM3 expression, which in turn decreases Aβ and tau burden.

It is generally accepted that Aβ deposition is one of the most representative pathological phenotypes for AD [25,26]. The Aβ fibrils augment oxidative stress and trigger inflammatory cascade, in turn accelerating cell death and causing fatal damage even to nearby neurons [27]. Interestingly, Aβ accumulates in both intracellular and extracellular spaces. A great body of evidence revealed that intracellular Aβ was produced within the endoplasmic reticulum (ER) and Golgi system or was derived from internalized extracellular Aβ [28,29]. In this regard, to relieve the entire burden of Aβ peptides, it is crucial to diminish both intra- and extracellular Aβ deposition and thereby minimize cellulogenic risk factors. Our study showed that there was a marked alleviation in Aβ deposition in both intra- and extracellular areas after KLDR treatment. It suggests that KLDR has a key role in improving intra- and extracellular Aβ burden in AD brain.

Then, a question remains about why such effects are induced under KLDR, rather than MLDR, regimen. A previous study explained that orthovoltage radiation exhibits higher X-ray absorption in tissues, along with more pronounced anti-inflammatory effects in surrounding organs [7]. Basically, photoelectric effects predominate at the relatively low X-ray energy range, indicating that the probability of photoelectric absorption decreases with increasing radiation energy [30,31]. These results imply that low-energy X-rays are more likely to induce biological effects in the targeted tissue than high-energy ones. Specifically, Amols et al. reported that at doses of 100-rad (1 Gy) or less, megavoltage photons (or electrons) showed radiobiological effectiveness (RBE) less than 0.8 compared to orthovoltage X-rays, and which might decrease significantly at lower doses. It indicates that the RBE of orthovoltage X-rays is at least 20% greater than that of the megavoltage radiation at 1-Gy dose or less [32].

In the present study, we employed the 3xTg-AD mouse model, which recapitulates both amyloid-β and tau pathology and reflects key features of AD. Its age-dependent progression enables evaluation of low-dose radiation therapy (LDRT) effects across disease stages, including amyloid deposition, tau pathology, neuroinflammation, and synaptic function [33]. However, as a familial AD-based model with relatively slow plaque development, it may not fully represent sporadic AD and can require longer experimental timelines [34]. Despite these limitations, it remains a valuable platform for integrated assessment of therapeutic effects. The AD model mice used in this study were 26–28 weeks old, an age at which neuroinflammation and Aβ/tau pathology begin to manifest [35], reflecting early-stage AD in clinical settings. At this stage, extracellular Aβ42 deposition forms amyloid plaques, while phosphorylated tau accumulates within neurons. Early microglial and astrocytic activation contributes to neuronal damage, synaptic loss, and subsequent cognitive decline [36,37]. In this study, we analyzed each of the factors as a function of radiation energy level and demonstrated the therapeutic efficacy of low-energy LDR (KLDR) in this disease process. Finally, with respect to AD-related transgenes expressed in the 3xTg-AD mouse model, LDR treatment did not alter their expression levels, APPswe, Tau P301L, and PS1. This finding suggests that the beneficial effects of LDR are unlikely to result from changes in transgene expression itself, but rather from modulation of downstream pathological processes, including neuroinflammation, amyloid pathology, and neuronal dysfunction.

Notably, our outcomes were achieved using a substantially lower radiation dose (0.6 Gy per fraction; total dose of 3 Gy) compared to that used in previous radiation studies (typically 2 Gy per fraction; total dose of 10 Gy), representing an approximately threefold reduction [8,11,14,15,16,38,39,40,41,42]. This difference highlights that therapeutic effects can be attained even at markedly reduced dose levels. In particular, our results suggest that modulation of AD pathology does not necessarily require higher radiation exposure, but may instead depend on appropriately optimized irradiation conditions, including energy and dose rate. From a translational perspective, the use of lower doses may also help minimize potential radiation-related risks while maintaining efficacy. These findings indicate that AD pathology can be improved at lower radiation doses, offering valuable evidence for optimizing LDR regimens. Therefore, these results provide important evidence that AD pathology can be improved at lower radiation doses and support the optimization of LDR regimens toward safer and more clinically feasible therapeutic strategies.

The radiation dose (Gy) applied in this study was determined through a combination of literature review and experimental validation. In a pioneering approach, a 2-Gy protocol (2 Gy per fraction × 5; total dose, 10 Gy) was adopted in LDRT studies for AD model mice [40,43]. Yang et al. evaluated both the 2-Gy protocol and a 0.6-Gy protocol (0.6 Gy per fraction × 5; total dose, 3 Gy) in AD model mice, demonstrating that the 0.6-Gy protocol produced therapeutic effects comparable to those of the 2-Gy protocol [42]. Subsequently, the 0.6-Gy protocol has been applied not only to AD [44] but also to other conditions, including osteoarthritis [45]. Based on these prior findings, together with our experimental validation, we determined that the 0.6-Gy protocol is appropriate for the present study.

KLDR irradiation is technically feasible for the human brain, despite the presence of cranial bone. The mass attenuation coefficients (μ/ρ, m^2^/kg) of 100 keV X-ray photon energy in water and bone are 1.707 × 10^−2^ and 1.855 × 10^−2^, respectively, indicating a maximum difference of approximately 8.76% at best [46]. This discrepancy is further reduced when 110 keV X-rays—slightly exceeding 100 keV—are utilized. Given an average skull thickness of 6.9 mm (range: 6.5–7.1 mm) [47], exponential attenuation analysis (I = I_0_·e^−μx^) indicates ~78.9% X-ray transmission, supporting sufficient energy delivery to the brain during whole-brain LDR in humans.

This study was conducted using male mice. Generally, in 3xTg-AD mice, females exhibit more severe disease progression in terms of Aβ/tau pathology and neuroinflammation compared to males [35]. Likewise, lecanemab—a newly approved drug for Alzheimer’s disease (AD)—has shown limited efficacy in female patients [48]. Given these sex-dependent differences in disease progression and severity, we considered that mixing male and female mice within the same experimental group could introduce substantial biological variability. Accordingly, the present study focused on male mice to ensure experimental consistency and reduce potential confounding effects. In future studies, we plan to systematically investigate whether the therapeutic efficacy of LDRT differs between sexes or remains consistent regardless of sex, thereby addressing sex-specific responses to LDRT.

Another limitation of the present study is that long-term safety was not evaluated. Although the current study demonstrated the superior therapeutic efficacy of KLDR compared with MLDR in 3xTg-AD mice, the experimental design primarily focused on therapeutic outcomes rather than long-term safety assessment. Therefore, potential delayed effects associated with LDR were not examined. Given that long-term safety is a critical consideration for clinical translation, future studies involving extended follow-up periods and comprehensive safety evaluations will be necessary to further establish the safety profile of KLDR.

## 4. Materials and Methods

### 4.1. Animals and Group Assignment

A total of 16 male homozygous 3xTg-AD transgenic mice [B6;129-Tg(APPSwe,tauP301L)1Lfa *Psen1^tm1Mpm^*/Mmjax] (Jackson Laboratory, Bar Harbor, ME, USA) aged 26–28 weeks old were used in the study. All mice were housed 4 to 5 per cage at room temperature with an alternating 12/12 h light/dark cycle and free access to food and water. They were assigned to the following groups: Sham (n = 5), KLDR (n = 6), and MLDR (n = 5) groups. Sham radiation is defined by a radiation regime in which LDR is irradiated with 0 Gy dose in each treatment.

### 4.2. Cranial LDR Procedure

Mice were anesthetized with an intraperitoneal injection of Zoletil (2.5 mg/kg, Virbac, Carros, France) using a 0.5 mL insulin syringe with an ultra-fine needle (BD Biosciences, San Jose, CA, USA) and were placed on an immobilizer before irradiation. The head irradiation was performed at a dose of 0.6 Gy twice a week for two and a half weeks, a total of 5 times (total dose, 3 Gy in 5 fractions), using an in vivo X-ray irradiation system (RS320, Xstrahl Ltd., Walsall, West Midlands, UK) for the KLDR group (110 kV, 10 mA, 0.6 Gy/min), or a linear accelerator (RapidArc, Varian Medical Systems, Palo Alto, CA, USA) for the MLDR group (6 MV, 0.6 Gy/min). In the sham group, all procedures were conducted identically to those in the LDR treatment groups (KLDR and MLDR), except that irradiation was delivered at 0 Gy.

### 4.3. Cognitive Behavioral Tests

#### 4.3.1. Passive Avoidance (PA) Test

The passive avoidance test was performed using a test apparatus (LE872, Panlab, Barcelona, Spain): 250 (W) × 250 (D) × 240 (H) mm white compartment and 195 × 108 × 120 mm black compartment. For adaptation training, the animals were placed in the light compartment for 10 min and allowed to freely enter and exit both compartments for 10 min. On the training day, each mouse was allowed to move freely into the dark chamber, where then the mouse was given 0.3 mA electrical shock in 5 s. On the next day, the mice were re-tested in the same way, and the step-through latency before re-entering the dark side was measured up to 300 s using SHUTAVOID software (v1.9, Panlab, Barcelona, Spain).

#### 4.3.2. Novel Object Recognition (Nor) Test

Mice were first acclimated to a 45 cm cube-shaped test chamber on Day 1. The following day, they were exposed to two identical objects within the same environment to facilitate object familiarization through free exploration. Subsequently, one of the objects was replaced with a novel object. All behavior of the mice inside the chamber was video recorded. The trajectories of object exploration were analyzed with a SMART video tracking S/W (v3.0, PanLab Harvard Apparatus, Holliston, MA, USA). The PI and DI were used for quantifying the interest of AD model mice in the novel object. The formulas of the indices are: PI, Time spent on novel object/Time spent on novel object + Time spent on familiar; DI, Time spent on novel object—Time spent on familiar object/Time spent on novel object + Time spent on familiar.

### 4.4. Brain Tissue Preparation

AD model mice were sacrificed under isoflurane inhalation anesthesia (2.0–2.5% *v*/*v* in O_2_) using an isoflurane vaporizer (VetEquip, Livermore, CA, USA) at 9 weeks after the last treatment. They were transcardially perfused with phosphate-buffered saline (PBS) and brains were carefully dissected. The extracted brains were sagittally divided in half. One half was used for histological analysis, and the other half was used for mRNA or protein quantification. Each brain section was prepared in the horizontal plane at a thickness of 40 μm using a microtome (Leica VT1000S, Nussloch, Germany). In histological analyses, one or 2–3 brain slice tissues were used per mouse. Histological and quantitative analyses were performed in key brain areas closely related to cognitive function such as hippocampus [CA1, dorsal subiculum (dSub), dentate gyrus], cortex, and amygdala.

### 4.5. Histological Analyses

#### 4.5.1. Immunofluorescence Analysis

Free-floating brain sections were incubated overnight at 4 °C with the following primary antibodies: rat monoclonal glial fibrillary acidic protein (GFAP) (1:1000; 13-0300, Thermo Fisher Scientific Inc., Waltham, MA, USA), goat polyclonal ionized calcium binding adaptor molecule 1 (Iba-1) (1:250; ab5076, Abcam, Cambridge, UK), mouse monoclonal neuronal nuclei (NeuN) (1:100; MAB377, Sigma-Aldrich, St. Louis, MO, USA), mouse synaptophysin (SYN) (1:500; S5768, Sigma-Aldrich), mouse monoclonal Tau (HT7) (1:1000; MN1000, Thermo Fisher Scientific Inc.), mouse monoclonal phospho-Tau (AT180) (1:500, MN1040, Thermo Fisher Scientific Inc.). Subsequently, Alexa Fluor-conjugated secondary antibodies were applied for 1 h at room temperature. Stained sections were mounted and imaged using a slide scanner (Axio Scan, Carl Zeiss, Jena, Germany).

#### 4.5.2. Immunohistochemical Analysis

For anti-6E10 and anti-4G8 staining, the sections were incubated at 4 °C on a shaker overnight with the following primary antibodies: mouse monoclonal β-Amyloid (6E10; 1:200; 803001, Biolegend, San Diego, CA, USA) (4G8; 1:200; 800701, Biolegend). Afterwards, the sections were incubated with #4 Linking Reagent and #5 Labeling Reagent for 20 min each on a shaker at room temperature (929501, Biolegend), and then mounted on slides. All stained sections were photographed under a microscope slide scanner (Axio scan, Carl Zeiss).

#### 4.5.3. Thioflavin S Staining

Free-floating sections were left to air dry overnight at room temperature and were then incubated for 10 min in 1% Thio-S (T1892, Sigma-Aldrich). After washing in 70% and 50% alcohol and then in distilled water, the sections were mounted on slides. All stained sections were photographed under a microscope slide scanner (Axio scan, Carl Zeiss).

### 4.6. Quantitative Molecular Analyses

#### 4.6.1. RT-qPCR

cDNA synthesis was accomplished using 1 μg of isolated RNA and ReverTra Ace^TM^ qPCR RT Master Mix (FSQ-201, TOYOBO, Osaka, Japan). qRT-PCR was performed using 2X Fast Q-PCR Master Mix (TQ1210, SMOBIO, Hsinchu, Taiwan) in a CFX Connect Real-Time PCR Detection System (1855201, Bio-Rad, Hercules, CA, USA). The following sets of sense/antisense primers were used: (i) F, 5′-CAGGCTCTGGAGAACAGCACAT-3′ and R, 5′-GACACATGCTGGCTTCTCTTCC-3′ for TGF-α; (ii) F, 5′-CAGAGCTGCGCTTGCAGAG-3′ and R, 5′-GTCAGCAGCCGGTTACCAAG-3′ for TGF-β; (iii) F, 5′-ATGCTGCCTGCTCTTACTGA-3′ and R, 5′-GCAGCTCTAGGAGCATGTGG-3′ for IL-10; (iv) F, 5′-GAGGATACCACTCCCAACAGACC-3′ and R, 5′-AAGTGCATCATCGTTGTTCATACA-3′ for Interleukin 6 (IL-6); (v) F, 5′-TCGTAGCAAACCACCAAGTG-3′ and R, 5′-ATATAGCAAATCGGCTGACG-3′ for TNF-α; (vi) F, 5′-CAGGCTCCGAGATGAACAAC-3′ and R, 5′-GGTGGAGAGCTTTCAGCTCATA-3′ for IL-1β; (vii) F, 5′-TCCCACTCGCACAGCAG-3′ and R, 5′-CATCAGTGGGTACCTCCAGC-3′ for APPswe; (viii) F, 5′-GGAAGATGTGACAGCACCCCT-3′ and R, 5′-CCTGCTTCTTCAGCTGTGGT-3′ for TauP301L; (ix) F, 5′-ACAGGTGCTACAAGGTCATCC-3′ and R, 5′-TGTAACGTAGTCCACGGCGC-3′ for PS1M146V; and (x) F, 5′-TGGCACAGTCAAGGCTGAGA-3′ and R, 5′-CTTCTGAGTGGCAGTGATGG-3′ for GAPDH. Gene expression analysis was performed using CFX Maestro Software (v2.3; Bio-Rad), and relative gene expression levels were normalized to GAPDH and calculated using the 2^−ΔΔCt^ method.

#### 4.6.2. ELISA

The concentrations of Aβ42 (DAB142, R&D Systems, Minneapolis, MN, USA), Aβ40 (DAB140B, R&D Systems), TGF-α (CSB-E07290m, Cusabio, Wuhan, China), TGF-β (ab119557, Abcam), IL-10 (ab100710, Abcam), TNF-α (ab108910, Abcam), IL-6 (ab100713, Abcam), and IL-1β (ab197742, Abcam) in mouse brain homogenates were quantified using commercially available ELISA kits according to the manufacturers’ instructions. Absorbance was measured at 450 nm using a SpectraMax Plus 384 microplate reader (Molecular Devices, San Jose, CA, USA), and concentrations were determined from standard curves.

### 4.7. Statistical Analysis

Statistical analyses were conducted using GraphPad Prism 10.2.3 (GraphPad Software Inc., San Diego, CA, USA) and SPSS software ver. 26 (IBM, Armonk, NY, USA). Data produced in the study were represented as mean ± standard errors of mean (SEM). Differences between groups were analyzed using a one-way repeated measures analysis of variance (ANOVA), followed by a post hoc Tukey’s test or Holm–Sidak for multiple comparisons. Data, which were not evenly distributed and did not pass the normality test, were analyzed using the Friedman test with a post hoc Tukey’s test for multiple comparisons. *p* values of less than 0.05 were considered statistically significant.

## 5. Conclusions

This study is the first direct comparative evaluation of KLDR and MLDR in AD model mice, focusing on the amelioration of early-stage AD features. Our findings suggest that kilovoltage energy is a key factor of LDR efficacy and support KLDR as a promising approach for AD treatment. Further studies are warranted to fine-tune this regimen and advance toward an optimized therapeutic protocol. Notably, variations in dose rate can lead to distinct biological outcomes, and identifying the optimal dose rate for improving AD remains an important research challenge. In general, lower dose rates allow more time for the repair of DNA damage and cellular injury [49]. Importantly, these findings provide a translational basis for the clinical application of KLDR, as kilovoltage X-ray systems are widely available and may allow safe and precise dose delivery in clinical settings. With further validation in large-animal models and well-designed clinical studies, KLDR could represent a feasible and scalable therapeutic strategy for patients with early-stage AD. Collectively, our results may contribute to optimizing LDRT protocol for AD and also inspire the development of novel radiation systems employing low-dose X-ray beams within optimized energy, dose ranges, and dose rates for the treatment of AD and other neurodegenerative disorders.

## Data Availability

The data presented in this study are openly available in the Supplementary Materials.

## Supplementary Materials

The following supporting information can be downloaded at: https://www.mdpi.com/article/10.3390/ijms27125458/s1.

## Abbreviations

The following abbreviations are used in this manuscript:

AD	Alzheimer’s disease
KLDR	kilovoltage low-dose radiation
MLDR	megavoltage low-dose radiation
LDR	low-dose radiation
LDRT	low-dose radiation therapy
PCR	polymerase chain reaction
ELISA	enzyme-linked immunosorbent assay
GFAP	glial fibrillary acidic protein
IL	interleukin
TGF	transforming growth factor
TNF	tumor necrosis factor
LPS	lipopolysaccharide
