# Supplementary material for: Kilovoltage Energy Significantly Enhances the Therapeutic Efficacy of Low-Dose Radiation in a 3xTg-AD Mouse Model of Alzheimer’s Disease
Source: Int J Mol Sci. 2026 Jun 17;27(12):5458. doi: 10.3390/ijms27125458 (PMC13300003; doi:10.3390/ijms27125458)
Supplement: Supplementary file 1 [file ijms-27-05458-s001.zip › Supple_Figures S1-S8.pdf]

Supplementary Figure S1.

A

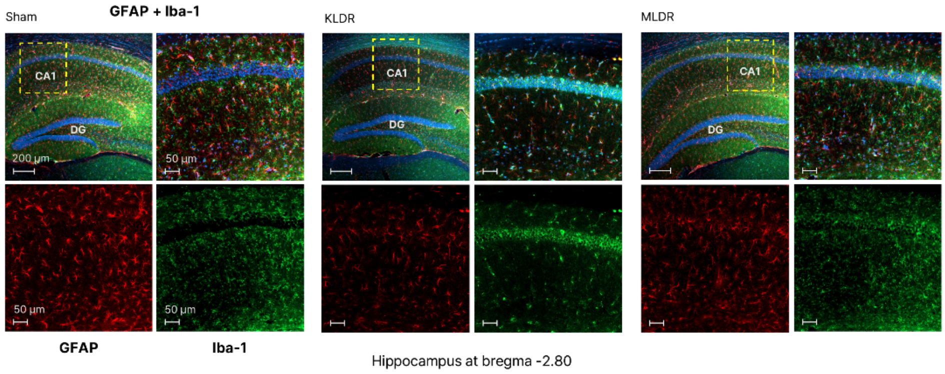

Hippocampus at bregma -2.80

A) Iba-1-positive and GFAP-positive cell counts in the CA1 region in the Sham, MLDR, and KLDR groups

Group	Iba-1-Positive Cell Count					GFAP-Positive Cell Count				
	Mean	SD	SEM	P value (vs. Sham)	P value (vs. MLDR)	Mean	SD	SEM	P value (vs. Sham)	P value (vs. MLDR)
**Sham**	1049	194.5	61.5	-	-	1759	503.2	159.1	-	-
**KLDR**	662.9	110.7	28.58	<0.001***	<0.001***	1218	366.8	105.9	0.008**	0.01*
**MLDR**	963.7	191.2	57.66	0.24	-	1705	237.7	75.17	0.75	-

*, **, and *** denote statistical significance at p<0.05, p<0.01, and p<0.001, respectively.

B

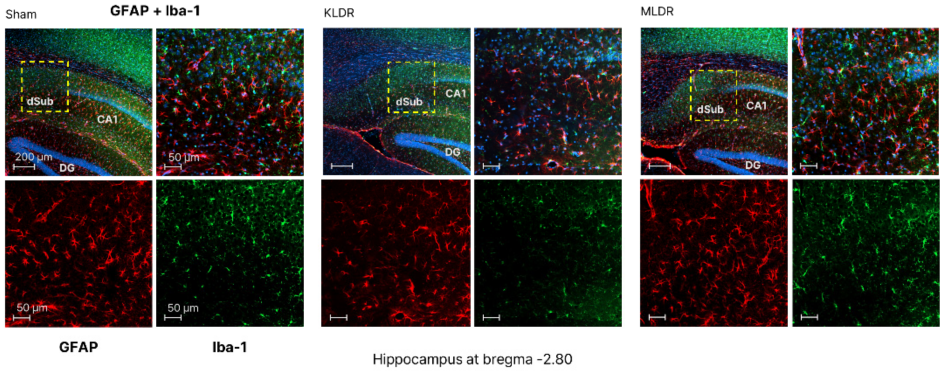

Hippocampus at bregma -2.80

B) Iba-1-positive and GFAP-positive cell counts in the dSub region in the Sham, MLDR, and KLDR groups

Group	Iba-1-Positive Cell Count					GFAP-Positive Cell Count				
	Mean	SD	SEM	P value (vs. Sham)	P value (vs. MLDR)	Mean	SD	SEM	P value (vs. Sham)	P value (vs. MLDR)
**Sham**	955.8	86.13	27.24	-	-	1619	367.9	110.9	-	-
**KLDR**	680.9	167.9	43.34	<0.001***	0.005**	1124	376.6	100.7	0.003**	0.03*
**MLDR**	868.9	151.6	45.71	0.18	-	1476	262.4	75.75	0.32	-

*, **, and *** denote statistical significance at p<0.05, p<0.01, and p<0.001, respectively.

(A) Immunofluorescence images of GFAP-positive astrocytes (red) and Iba-1-positive microglia (green) in the CA1 region of sham-, KLDR-, and MLDR-treated mice. Merged images and higher-magnification views are shown. Scale bars = 200 μm (low magnification) and 50 μm (high magnification).

(B) Immunofluorescence images of GFAP-positive astrocytes (red) and Iba-1-positive microglia (green) in the dorsal subiculum (dSub) of sham-, KLDR-, and MLDR-treated mice. Merged images and higher-magnification views are shown. Scale bars = 200 μm (low magnification) and 50 μm (high magnification).

Supplementary Figure S2.

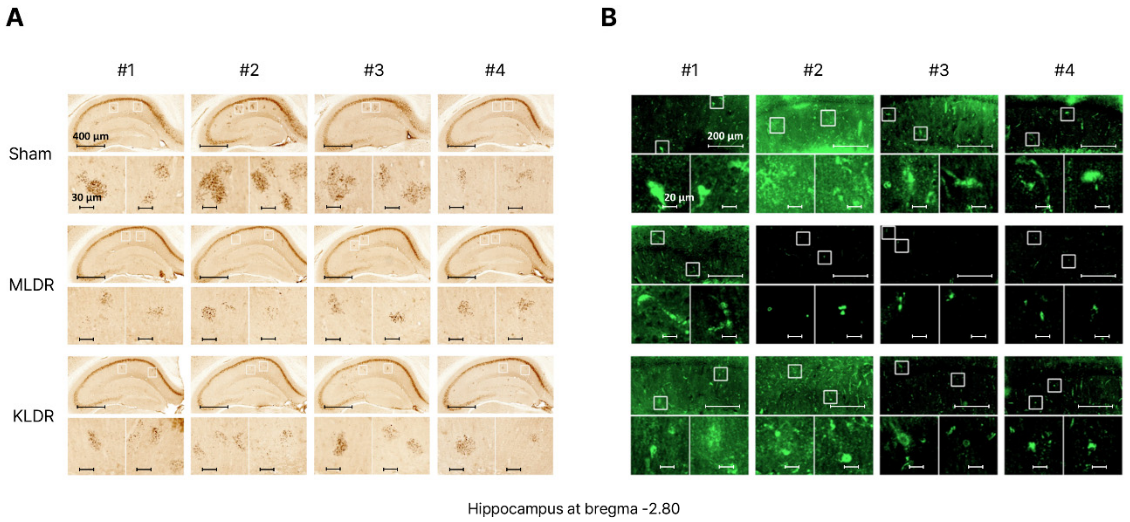

A, B) Hippocampal 4G8 and Thioflavin S intensity in the Sham, KLDR, and MLDR groups

Group	4G8 (A)					Thioflavin S (B)				
	Mean	SD	SEM	P value (vs. Sham)	P value (vs. MLDR)	Mean	SD	SEM	P value (vs. Sham)	P value (vs. MLDR)
**Sham**	31.52	21.42	10.71	-	-	40.10	22.61	11.31	-	-
**KLDR**	7.11	4.20	2.10	0.0475*	0.6253	9.05	7.67	3.84	0.0490*	0.3172
**MLDR**	14.88	5.85	2.93	0.1779	-	25.64	16.38	8.19	0.4020	-

* denotes statistical significance at p<0.05.

(A) 4G8-immunostained hippocampal sections at bregma −2.80 mm from animals in the Sham, MLDR, and KLDR groups. Insets show higher-magnification views of the boxed regions. (B) Thioflavin S-stained hippocampal sections and corresponding enlarged images of the boxed regions. Quantitative analyses of 4G8 and Thioflavin S staining are summarized in the table below.

Supplementary Figure S3.

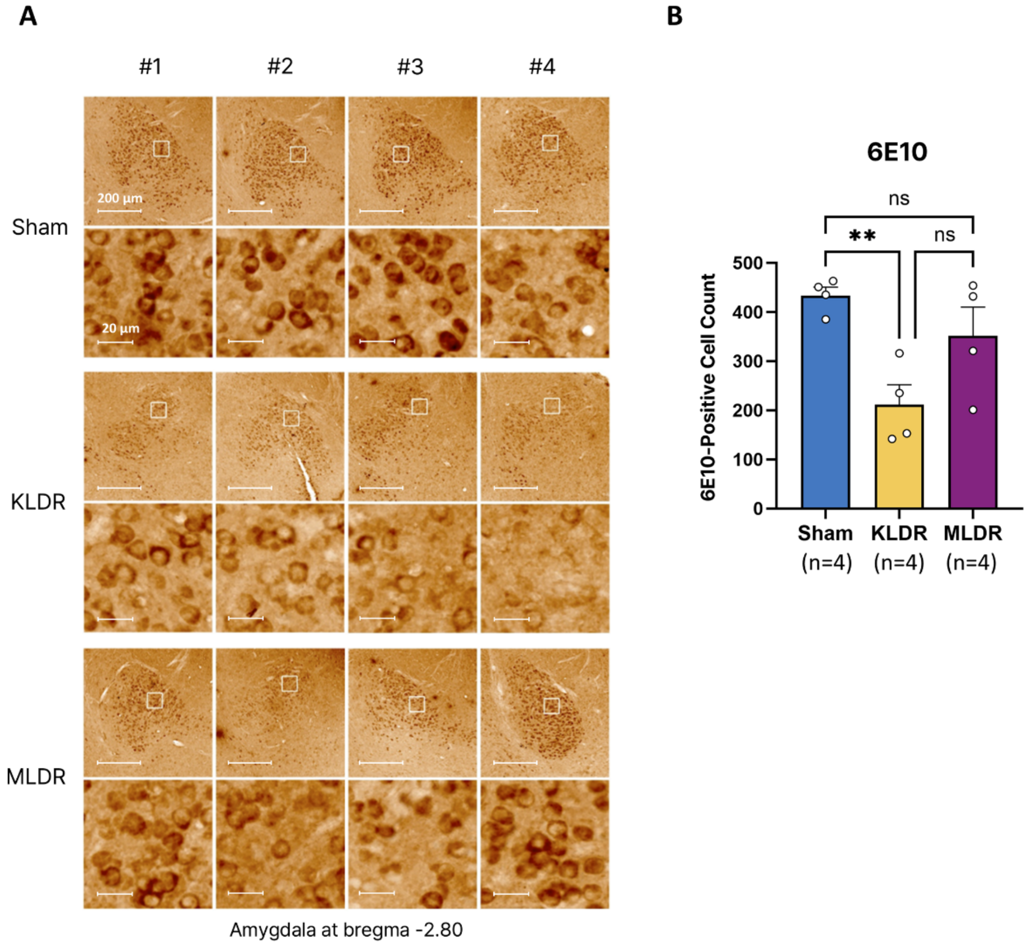

A, B) 6E10-positive cell counts in the amygdala in the Sham, KLDR, and MLDR groups

Group	Amygdala				
	Mean	SD	SEM	P value (vs. Sham)	P value (vs. MLDR)
**Sham**	433.50	34.31	17.15	-	-
**KLDR**	211.50	81.09	40.54	0.0086**	0.075
**MLDR**	352.00	116.30	58.14	0.3322	-

** denotes statistical significance at p<0.01.

Immunohistochemical analysis was performed in the amygdala slices at bregma -2.80 using a monoclonal β-Amyloid antibody (6E10) (A). The quantitative analysis showed that the number of 6E10-positive cells was significantly

reduced in the KLDR group (vs. sham, p<0.01), but MLDR group exhibited minimal effects in reducing intracellular Aβ deposition (vs. sham, p=0.3322; B). ** denotes statistical significance at p<0.01. NS, not significant

Supplementary Figure S4.

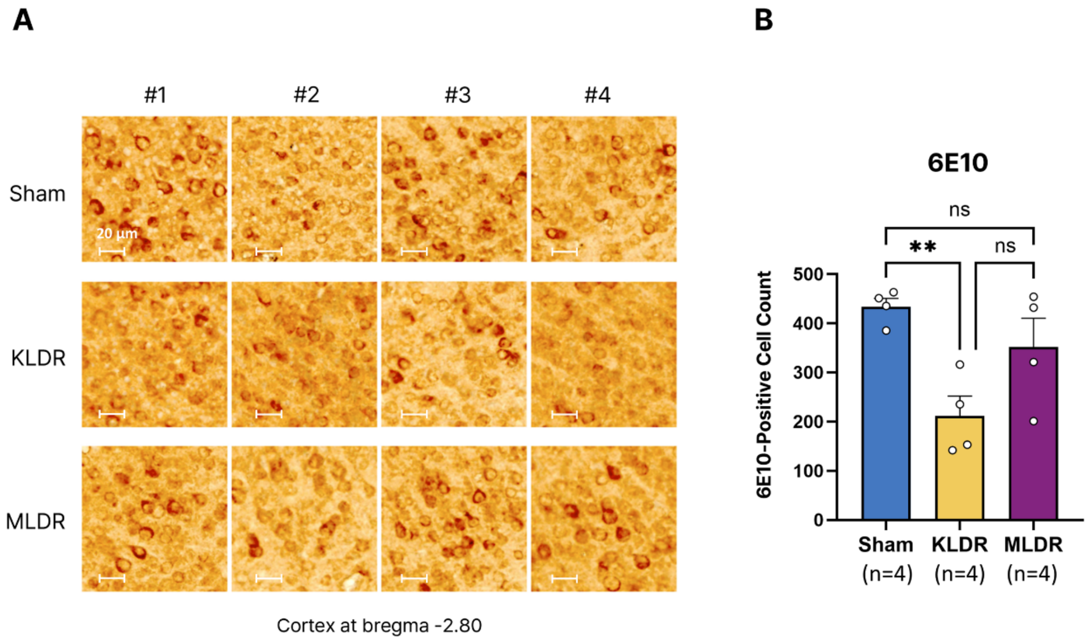

A, B) 6E10-positive cell counts in the cortex in the Sham, KLDR, and MLDR groups

**Group**	**Cortex**				
	Mean	SD	SEM	P value (vs. Sham)	P value (vs. MLDR)
**Sham**	126.80	19.74	9.87	-	-
**KLDR**	69.00	13.78	6.89	0.0009***	0.005**
**MLDR**	113.80	11.79	5.89	0.42	-

** and *** denote statistical significance at p<0.01 and p<0.001, respectively.

Immunohistochemical analysis was performed in the cortical slices at bregma -2.80 using a monoclonal β-Amyloid antibody (6E10) (A). The number of 6E10-positive cells was significantly reduced in the KLDR group (vs. sham, p<0.001), which also showed a significant difference compared to the MLDR group (p<0.05; B). In

contrast, MLDR group exhibited minimal effects in reducing intracellular Aβ deposition (vs. sham, p=0.4206; B). * and *** denote statistical significance at p<0.05 and p<0.001, respectively. NS, not significant

Supplementary Figure S5

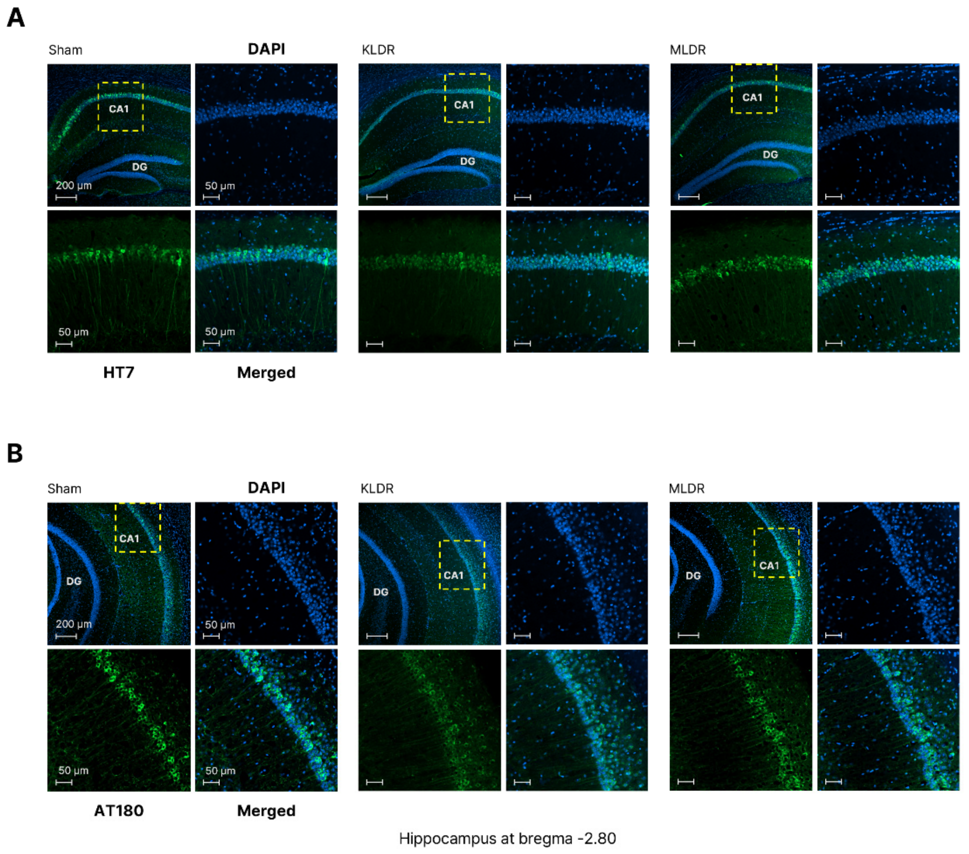

A, B) The fluorescent intensity of HT7 and AT180 in the CA1 region in the Sham, MLDR, and KLDR groups

Group	HT7 (A)					AT180 (B)				
	Mean	SD	SEM	P value (vs. Sham)	P value (vs. MLDR)	Mean	SD	SEM	P value (vs. Sham)	P value (vs. MLDR)
**Sham**	157.1	29.54	9.34	-	-	137.3	14.82	4.68	-	-
**KLDR**	106.1	12.33	3.42	<0.001***	<0.001***	111.7	18.98	5.48	0.023*	0.022*
**MLDR**	153.6	23.31	7.03	0.72	-	135.4	27.1	8.17	0.835	-

* and *** denote statistical significance at p<0.05 and p<0.001, respectively.

(A) HT7 immunofluorescence staining in hippocampal sections at bregma −2.80 mm. The dashed boxes indicate the CA1 region, and enlarged views of the boxed areas are shown below. HT7 staining is shown in green and

DAPI nuclear staining in blue. (B) AT180 immunofluorescence staining in hippocampal sections at the same anatomical level. Enlarged images of the boxed CA1 regions are shown below. AT180 staining is shown in green and DAPI nuclear staining in blue. Quantitative analyses of HT7 and AT180 fluorescence intensities in the CA1 region are summarized in the table below.

Supplementary Figure S6

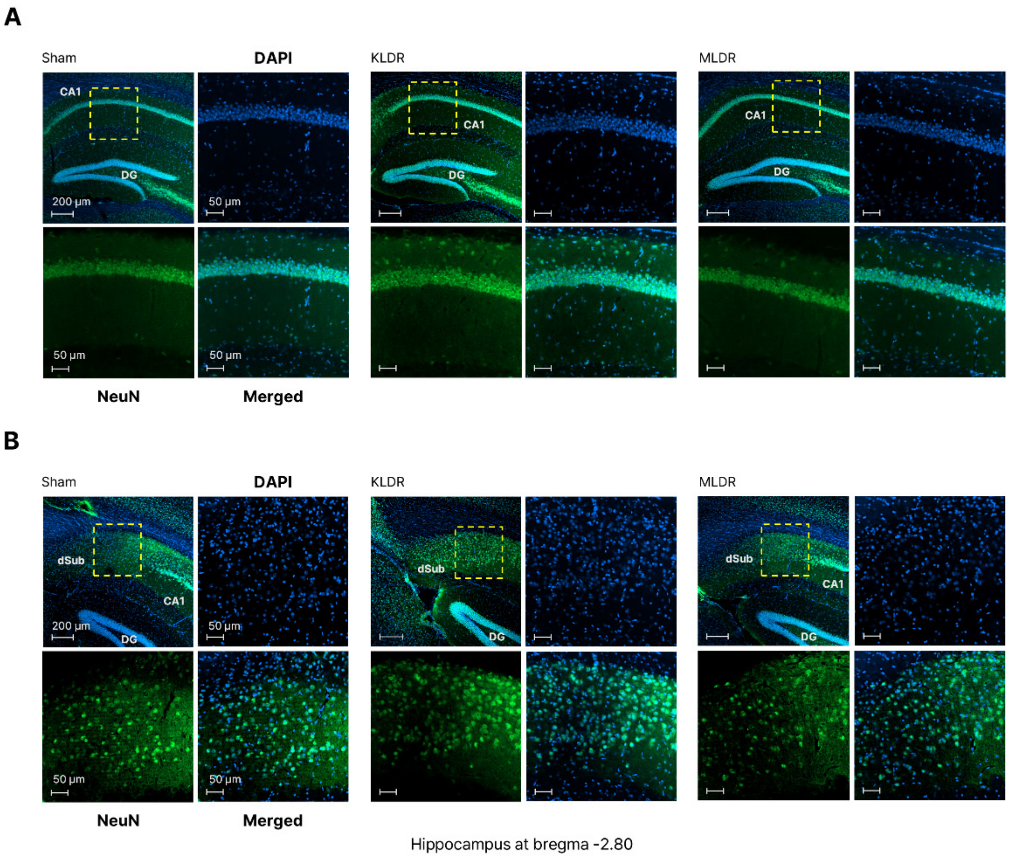

A, B) NeuN fluorescence intensity (CA1) and NeuN-positive cell count (dSub) in the Sham, MLDR, and KLDR groups

Group	NeuN Fluorescence Intensity (CA1)					NeuN-Positive Cell Count (dSub)				
	Mean	SD	SEM	P value (vs. Sham)	P value (vs. MLDR)	Mean	SD	SEM	P value (vs. Sham)	P value (vs. MLDR)
**Sham**	87.85	14.68	4.24	-	-	13322	3936	1187	-	-
**KLDR**	109.3	23.08	6.17	0.02*	0.025*	20575	5539	1536	0.002**	0.04*
**MLDR**	88.41	12.09	3.49	0.919	-	16041	4308	1244	0.17	-

* and ** denote statistical significance at p<0.05 and p<0.01, respectively.

(A) NeuN-stained hippocampal sections at bregma −2.80 mm. The dashed boxes indicate the CA1 region, and enlarged views of the boxed areas are shown below. (B) NeuN-stained hippocampal sections highlighting the dentate subgranular zone (dSub), with enlarged views of the boxed regions shown below. Green, NeuN immunofluorescence; blue, DAPI. Quantitative analyses of NeuN fluorescence intensity in the CA1 region and NeuN-positive cell counts in the dSub are presented in the table below.

Supplementary Figure S7

A

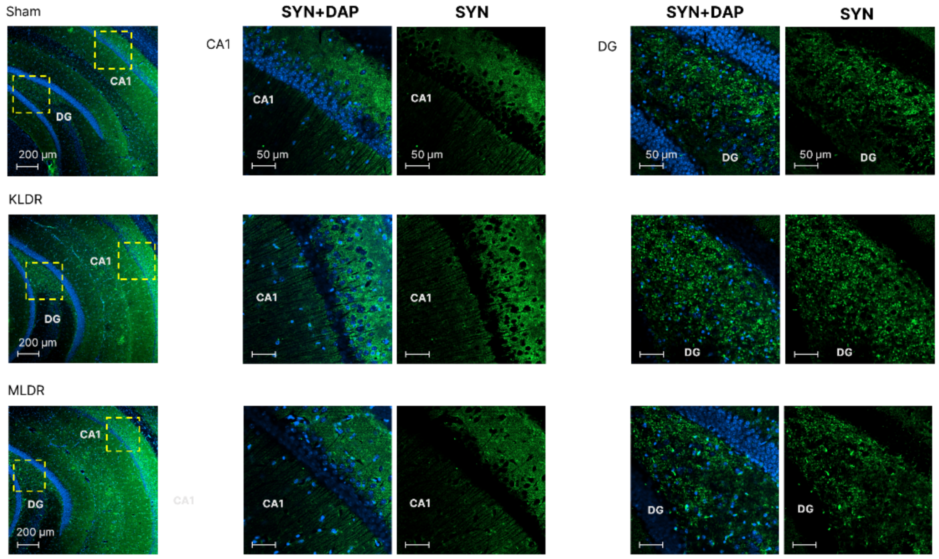

Hippocampus at bregma -2.80

A) SYN fluorescence intensity (CA1, DG) in the Sham, MLDR, and KLDR groups

Group	SYN (CA1)					SYN (DG)				
	Mean	SD	SEM	P value (vs. Sham)	P value (vs. MLDR)	Mean	SD	SEM	P value (vs. Sham)	P value (vs. MLDR)
**Sham**	109.4	13.72	3.96	-	-	109.9	13.66	4.19	-	-
**KLDR**	137.8	23.61	6.09	<0.001***	0.02*	131.7	16.45	4.39	0.002**	<0.001***
**MLDR**	119.0	13.6	3.63	0.18	-	99.05	13.81	3.98	0.09	-

* , **, and *** denote statistical significance at p<0.05, p<0.01, p<0.001, respectively.

(A) SYN-stained hippocampal sections at bregma −2.80 mm. Dashed boxes indicate the CA1 region and dentate gyrus (DG), and enlarged views of the corresponding regions are shown on the right. Green, SYN

immunofluorescence; blue, DAPI. Quantitative analyses of SYN fluorescence intensity in the CA1 region and DG are presented in the table below.

Supplementary Figure S8

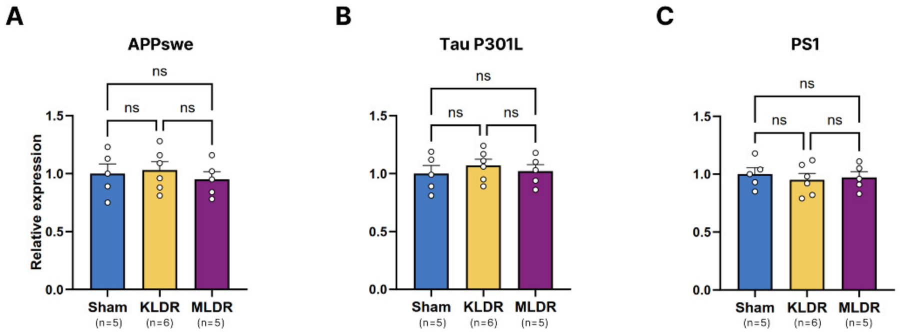

A, B, C) Transgene expression levels in the Sham, MLDR, and KLDR groups

Group	APPswe (A)					Tau P301L (B)					PS1 (C)				
	Mean	SD	SEM	P value (vs. Sham)	P value (vs. MLDR)	Mean	SD	SEM	P value (vs. Sham)	P value (vs. MLDR)	Mean	SD	SEM	P value (vs. Sham)	P value (vs. MLDR)
**Sham**	1	0.19	0.08497	-	-	1	0.1572	0.07029	-	-	1	0.1239	0.05541	-	-
**KLDR**	0.95	0.15	0.06708	0.9564	0.7333	1.022	0.1266	0.0566	0.6899	0.8375	0.972	0.111	0.04964	0.7998	0.9607
**MLDR**	1.03	0.1782	0.07276	0.8933	-	1.07	0.1322	0.05398	0.9660	-	0.9517	0.1342	0.0548	0.9328	-

(A) APPswe, (B) Tau P301L, and (C) PS1 expression levels normalized to the Sham group. Individual data points are overlaid on bar graphs showing mean ± SEM. Quantitative analyses are presented in the table below. No significant differences in APPswe, Tau P301L, or PS1 expression levels were observed among the Sham, MLDR, and KLDR groups
