# Supplementary material for: Kilovoltage Energy Significantly Enhances the Therapeutic Efficacy of Low-Dose Radiation in a 3xTg-AD Mouse Model of Alzheimer’s Disease
Source: Int J Mol Sci. 2026 Jun 17;27(12):5458. doi: 10.3390/ijms27125458 (PMC13300003; doi:10.3390/ijms27125458)
Supplement: Supplementary file 1 [file ijms-27-05458-s001.zip › Supple_Table S1.pdf]

Supplementary Table S1

1. The latency in the acquisition and retention phases in the following groups: Sham, KLDR, and MLDR

(1) Week 0 (baseline)

Group	Acquisition Phase (sec)			Retention Phase (sec)		
	Mean	SD	SEM	Mean	SD	SEM
Sham	19	8.77	3.92	110	10.22	4.57
KLDR	23.33	4.37	1.78	104.67	12.88	5.26
MLDR	21.8	8.87	3.97	106.2	11.86	5.3

(2) Week 7

Group	Acquisition (sec)			Retention (sec)		
	Mean	SD	SEM	Mean	SD	SEM
Sham	18.2	6.22	2.78	71.4	7.99	3.57
KLDR	15.67	1.75	0.71	94.83	14.18	5.79
MLDR	19.8	5.85	2.62	89.4	12.6	5.64

(3) Week 12

Group	Acquisition (sec)			Retention (sec)		
	Mean	SD	SEM	Mean	SD	SEM
Sham	57.4	16.64	7.44	55.6	7.92	3.54
KLDR	66.67	10.8	4.41	104.17	11.7	4.78
MLDR	68.4	16.86	7.54	85.2	20.17	9.02

2. The latency difference between the acquisition and retention phases in the following groups: Sham, KLDR, and MLDR

Group	Week 0					Week 7					Week 12				
	Mean	SD	SEM	P value (vs. Sham)	P value (vs. MLDR)	Mean	SD	SEM	P value (vs. Sham)	P value (vs. MLDR)	Mean	SD	SEM	P value (vs. Sham)	P value (vs. MLDR)
Sham	91	17.23	7.71	-	-	53.2	6.34	2.83	-	-	-1.8	15.4	6.89	-	-
KLDR	81.33	10.29	4.2	0.63	0.70	79.17	14.09	5.75	0.005**	0.17	37.5	11.33	4.62	0.005**	0.11
MLDR	84.4	15.27	6.83	0.54	-	69.6	10.11	4.52	0.07	-	16.8	21.63	9.67	0.09	-

** denotes statistical significance at $p<0.01$.

3. The Preference Index in the following groups: Sham, KLDR, and MLDR

Group	Week 0					Week 7					Week 12				
	Mean	SD	SEM	P value (vs. Sham)	P value (vs. MLDR)	Mean	SD	SEM	P value (vs. Sham)	P value (vs. MLDR)	Mean	SD	SEM	P value (vs. Sham)	P value (vs. MLDR)
Sham	0.76	0.09	0.04	-	-	0.56	0.22	0.10	-	-	0.15	0.16	0.07	0.07	-
KLDR	0.75	0.10	0.04	0.96	0.85	0.51	0.13	0.05	0.95	0.77	0.48	0.04	0.02	0.029*	0.17
MLDR	0.75	0.14	0.06	1.00	-	0.43	0.19	0.09	0.91	-	0.36	0.10	0.05	0.14	-

* denotes statistical significance at p<0.05.

4. The Discrimination Index in the following groups: Sham, KLDR, and MLDR

Group	Week 0					Week 7					Week 12				
	Mean	SD	SEM	P value (vs. Sham)	P value (vs. MLDR)	Mean	SD	SEM	P value (vs. Sham)	P value (vs. MLDR)	Mean	SD	SEM	P value (vs. Sham)	P value (vs. MLDR)
Sham	0.52	0.19	0.08	-	-	0.13	0.45	0.20	-	-	-0.71	0.32	0.14	-	-
KLDR	0.51	0.21	0.08	0.96	0.85	0.01	0.25	0.10	0.95	0.78	-0.05	0.08	0.03	0.027*	0.16
MLDR	0.50	0.29	0.13	1.00	-	-0.13	0.38	0.17	0.91	-	-0.28	0.21	0.09	0.14	-

* denotes statistical significance at p<0.05.
