# Supplementary material for: Kilovoltage Energy Significantly Enhances the Therapeutic Efficacy of Low-Dose Radiation in a 3xTg-AD Mouse Model of Alzheimer’s Disease
Source: Int J Mol Sci. 2026 Jun 17;27(12):5458. doi: 10.3390/ijms27125458 (PMC13300003; doi:10.3390/ijms27125458)
Supplement: Supplementary file 1 [file ijms-27-05458-s001.zip › Supple_Table S2.pdf]

Supplementary Table S2

1. The mRNA levels of pro-inflammatory cytokines (TNF-α, IL-6, and IL-1β) in the sham, KLDR, and MLDR groups

(RT-qPCR; Region, Hippocampus)

Group	TNF-α					IL-6					IL-1β				
	Mean	SD	SEM	P value (vs. Sham)	P value (vs. MLDR)	Mean	SD	SEM	P value (vs. Sham)	P value (vs. MLDR)	Mean	SD	SEM	P value (vs. Sham)	P value (vs. MLDR)
Sham	1.00	0.33	0.15	-	-	1.00	0.31	0.16	-	-	1	0.1786	0.07988	-	-
KLDR	0.05	0.02	0.01	0.0077**	0.019*	0.66	0.50	0.25	0.5497	0.6687	0.5121	0.1838	0.07506	0.0015**	0.0115*
MLDR	0.85	0.51	0.25	0.79	-	0.93	0.61	0.31	0.9735	-	0.8819	0.1688	0.07551	0.8819	-

* and ** denote statistical significance at $p<0.05$ and $p<0.01$, respectively.

2. The protein levels of pro-inflammatory cytokines (TNF-α, IL-6, and IL-1β) in the sham, KLDR, and MLDR groups

(ELISA; Region, Hippocampus)

Group	TNF-α					IL-6					IL-1β				
	Mean	SD	SEM	P value (vs. Sham)	P value (vs. MLDR)	Mean	SD	SEM	P value (vs. Sham)	P value (vs. MLDR)	Mean	SD	SEM	P value (vs. Sham)	P value (vs. MLDR)
Sham	2.77	0.6165	0.2757	-	-	2.006	0.2655	0.1188	-	-	2.97	0.4876	0.2181	-	-
KLDR	1.634	0.2661	0.1086	0.0116*	0.0378*	1.587	0.2941	0.1201	0.0653	0.4956	1.757	0.3985	0.1627	0.0008***	0.0438*
MLDR	2.558	0.7088	0.317	0.8165	-	1.782	0.2703	0.1209	0.4337	-	2.432	0.3358	0.1502	0.1355	-

* and *** denote statistical significance at p<0.05 and p<0.001, respectively.

3. The mRNA levels of anti-inflammatory cytokines (TGF-α, TGF-β, IL10) in the sham, KLDR, and MLDR groups (RT-qPCR; Region, Hippocampus)

Group	TGF-α					TFG-β					IL-10				
	Mean	SD	SEM	P value (vs. Sham)	P value (vs. MLDR)	Mean	SD	SEM	P value (vs. Sham)	P value (vs. MLDR)	Mean	SD	SEM	P value (vs. Sham)	P value (vs. MLDR)
Sham	1	0.1608	0.0719	-	-	1	0.1761	0.07874	-	-	0.998	0.1858	0.08309	-	-

KLDR	1.2	0.102	0.04163	0.0723	0.4645	1.352	0.1376	0.05618	0.0046**	0.0366*	1.072	0.1566	0.06395	0.7784	0.9820
MLDR	1.1	0.1449	0.06481	0.4934	-	1.1	0.1279	0.05718	0.5485	-	1.052	0.1963	0.08777	0.8828	-

* and ** denote statistical significance at p<0.05 and p<0.01, respectively.

4. The protein levels of anti-inflammatory cytokines (TGF-α, TGF-β, IL10) in the sham, KLDR, and MLDR groups (ELISA; Region, Hippocampus)

Group	TGF-α					TFG-β					IL-10				
	Mean	SD	SEM	P value (vs. Sham)	P value (vs. MLDR)	Mean	SD	SEM	P value (vs. Sham)	P value (vs. MLDR)	Mean	SD	SEM	P value (vs. Sham)	P value (vs. MLDR)
Sham	1.7	0.4524	0.2023	-	-	3.4	1.035	0.4627	-	-	1.498	0.2228	0.09962	-	-
KLDR	2.9	0.9877	0.4032	0.0441*	0.0371*	5.305	0.9917	0.4049	0.0154*	0.0176*	1.562	0.2215	0.09042	0.8819	0.9978
MLDR	1.658	0.5607	0.2507	0.9955	-	3.442	0.8326	0.3724	0.8874	-	1.57	0.2124	0.09497	0.8633	-

* denotes statistical significance at p<0.05.
