# Supplementary material for: Kilovoltage Energy Significantly Enhances the Therapeutic Efficacy of Low-Dose Radiation in a 3xTg-AD Mouse Model of Alzheimer’s Disease
Source: Int J Mol Sci. 2026 Jun 17;27(12):5458. doi: 10.3390/ijms27125458 (PMC13300003; doi:10.3390/ijms27125458)
Supplement: Supplementary file 1 [file ijms-27-05458-s001.zip › Supple_Table S3.pdf]

Supplementary Table S3

1. The ELISA quantification of hippocampal Aβ40 and Aβ42 levels in the Sham, KLDR, and MLDR groups

Group	Aβ40					Aβ42				
	Mean	SD	SEM	P value (vs. Sham)	P value (vs. MLDR)	Mean	SD	SEM	P value (vs. Sham)	P value (vs. MLDR)
Sham	8.46	0.54	0.24	-	-	4.58	0.69	0.31	-	-
KLDR	6.57	0.5	0.2	0.001**	0.38	3.62	0.37	0.15	0.04*	0.6
MLDR	6.94	0.89	0.4	0.006**	-	3.79	0.55	0.25	0.08	-

* and ** denote statistical significance at $p<0.05$ and $p<0.01$, respectively.
